# Fluorescence-line-selective soft X-ray absorption spectroscopy: a novel approach to element-specific electronic structure analysis

**DOI:** 10.1107/S1600577526000615

**Published:** 2026-02-18

**Authors:** Kota Naito, Nobuo Nakajima, Shigetomo Shiki

**Affiliations:** a Faculty of Science Hiroshima University 1-3-1 Kagamiyama Higashi-Hiroshima Hiroshima739-8526 Japan; b Graduate School of Advanced Science and Engineering Hiroshima University 1-3-1 Kagamiyama Higashi-Hiroshima Hiroshima739-8526 Japan; c National Institute of Advanced Industrial Science and Technology 807-1 Shuku-machi Tosu Saga841-0052 Japan; Advanced Photon Source, USA

**Keywords:** soft X-ray XAS, fluorescence-line-selective XAS, superconducting tunnel junction

## Abstract

A novel approach to soft X-ray fluorescence-yield absorption spectroscopy is presented using a superconducting tunnel junction (STJ) X-ray detector, a new type of detector for the soft X-ray region. The STJ detector offers superior energy resolution compared with silicon drift detectors and higher detection efficiency than grating-based spectrometers, both of which are widely used in soft X-ray spectroscopy. The STJ detector can simultaneously detect multiple fluorescence lines in a single measurement, even on a bending-magnet beamline, which enables *medium*-energy-resolution fluorescence detected X-ray absorption spectroscopy (XAS) without the need for large-scale emission spectrometers. Using these characteristics, the Ti *L*α/*L*ℓ XAS and O *K*α XAS of SrTiO_3_ were measured, where Ti *L*ℓ XAS are expected to reflect the intrinsic Ti 3*d* electronic states without being affected by orbital anisotropy, providing a more accurate picture of the transition-metal electronic structure. These results demonstrate that the STJ detector is effective for probing anion electronic states of carbides, nitrides and oxides.

## Abstract 2

Fluorescence-line-selective soft X-ray spectroscopy using a superconducting tunnel junction (STJ) detector was applied to probe the electronic states of strontium titanate, demonstrating the capability of the STJ detector for high-energy-resolution soft X-ray absorption spectroscopy.

## 1. Introduction

X-ray absorption spectroscopy (XAS) in the soft X-ray region, which includes the *K*-edges of light elements and the *L*-edges of 3*d* transition metals, is a powerful technique for probing the electronic states of carbides, nitrides and oxides (Hellgren *et al.*, 2001 ▸; Zhong *et al.*, 2012 ▸; Frati *et al.*, 2020 ▸). In this energy range, strong X-ray absorption makes transmission-mode measurements difficult, except when specialized approaches such as deposition onto thin polymer films are used (Chen *et al.*, 1995 ▸). Consequently, total electron yield (TEY) measurements are commonly employed. Because the photoexcited electrons generated by soft X-ray excitation have escape depths of only several tens of angstroms (Tanuma *et al.*, 1994 ▸), TEY is highly surface-sensitive and therefore requires careful surface cleaning to obtain reliable spectra (Woicik & Pianetta, 2023 ▸). In addition, TEY is not suitable for measurements under applied electric or magnetic fields, because electron detection is incompatible with such environments, and therefore cannot be used for *operando* studies of anion electronic states in electronic devices. In contrast, fluorescence yield detection allows measurements under external fields (Sakaki *et al.*, 2013 ▸). Because the escape depth of fluorescent X-rays is substantially greater than that of photoexcited electrons, fluorescence yield detection provides more bulk-sensitive information with only limited influence from surface conditions. Despite these advantages, silicon drift detectors commonly used in the hard X-ray region have limited energy resolution (Δ*E* ≃ 100 eV) (Guazzoni, 2010 ▸; Agostini *et al.*, 2025 ▸), making them unsuitable for soft X-ray XAS. This limitation highlights the need for a detector with high energy resolution, high sensitivity and high count-rate performance. Cryogenic detectors utilizing superconducting tunnel junctions (STJs) and microcalorimeters have attracted attention as practical soft X-ray spectrometers, as they offer higher energy resolution than semiconductor detectors and superior sensitivity than grating-based spectrometers.

STJ detectors offer energy-dispersive X-ray detection and, in particular, provide high energy resolution, high sensitivity and high-count-rate performance (Friedrich, 2006 ▸; Friedrich *et al.*, 2006 ▸; Shiki & Fujii, 2025 ▸), making them especially suitable for low-energy soft X-ray spectroscopy (Maeda *et al.*, 2020 ▸; Isomura & Kimoto, 2021 ▸). An STJ is a superconducting diode composed of a superconductor/insulator/superconductor trilayer structure (Josephson junction). As an X-ray detector, the STJ provides strong signal intensity and excellent energy resolution because its superconducting gap is three orders of magnitude smaller than that of semiconductors (Kurakado, 1982 ▸; Kurakado *et al.*, 1993 ▸; Frank *et al.*, 1998 ▸). When X-rays enter an STJ detector, the absorbed photons break Cooper pairs in the superconducting electrodes and generate quasiparticles. The number of quasiparticles is proportional to the photon energy, enabling energy-dispersive detection. These quasiparticles tunnel through the insulating barrier, producing a current pulse whose integrated charge reflects the incident photon energy. This charge is used as the detection signal in STJ based X-ray spectroscopy. Furthermore, the high sensitivity and energy resolution of STJ detectors enable measurements on a bending-magnet beamline, securing ample beam time.

In this study, the STJ detector was used to measure the Ti *L*-edge and O *K*-edge XAS of SrTiO_3_, a representative dielectric oxide. SrTiO_3_ has a cubic perovskite structure in which Ti atoms are covalently bonded to the center of the oxygen octahedra (Barrett, 1952 ▸; Scott, 1974 ▸; Müller & Burkard, 1979 ▸). Due to the experimental difficulties in the soft X-ray region, the electronic structure of oxygen in this material remains insufficiently understood. Recent reports of ferro­electricity in SrTiO_3_ thin films (Haeni *et al.*, 2004 ▸; Jang *et al.*, 2010 ▸; Xu *et al.*, 2020 ▸; Li *et al.*, 2021 ▸) highlight the need to understand the electronic states of not only cations but also anions.

## 2. Experimental

A 5% Nb-doped SrTiO_3_ (001) single crystal with a size of 10 mm × 5 mm × 0.5 mm (Crystal Base Co. Ltd, Japan) was used as the sample. Soft X-ray absorption spectroscopy of the Ti *L*-edge and O *K*-edge was performed in fluorescence yield mode using an STJ detector. The experiment was performed at room temperature on the bending-magnet beamline BL12A (S-path) of the Photon Factory (KEK-PF) (Ohigashi *et al.*, 2025 ▸). The beamline energy resolution was approximately 0.2 eV at 500 eV (*E*/Δ*E* = 3000). The incident X-rays were parallel to the surface normal of the sample. The STJ detector was installed on the vacuum chamber and positioned perpendicular to the sample. During the experiment, the chamber was maintained at a pressure of 1.7 × 10^−6^ Pa.

The STJ detector used in this study is publicly available through the AIST Nanocharacterization Facility (ANCF) of the National Institute of Advanced Industrial Science and Technology (AIST, 2020 ▸). Although commercial STJ detectors exist (*e.g.* STAR Cryoelectronics) and partial fluorescence yield (PFY) XAS demonstrations have been reported, their routine use at synchrotron beamlines appears to be limited. Our detector is therefore currently the only ready-to-use STJ system implemented for PFY XAS measurements on soft X-ray beamlines (BL-12A, BL-13A and BL-16A) at KEK-PF.

## 3. Results

### 3.1. Overview of the performance of the STJ detector

**Figure 1:**
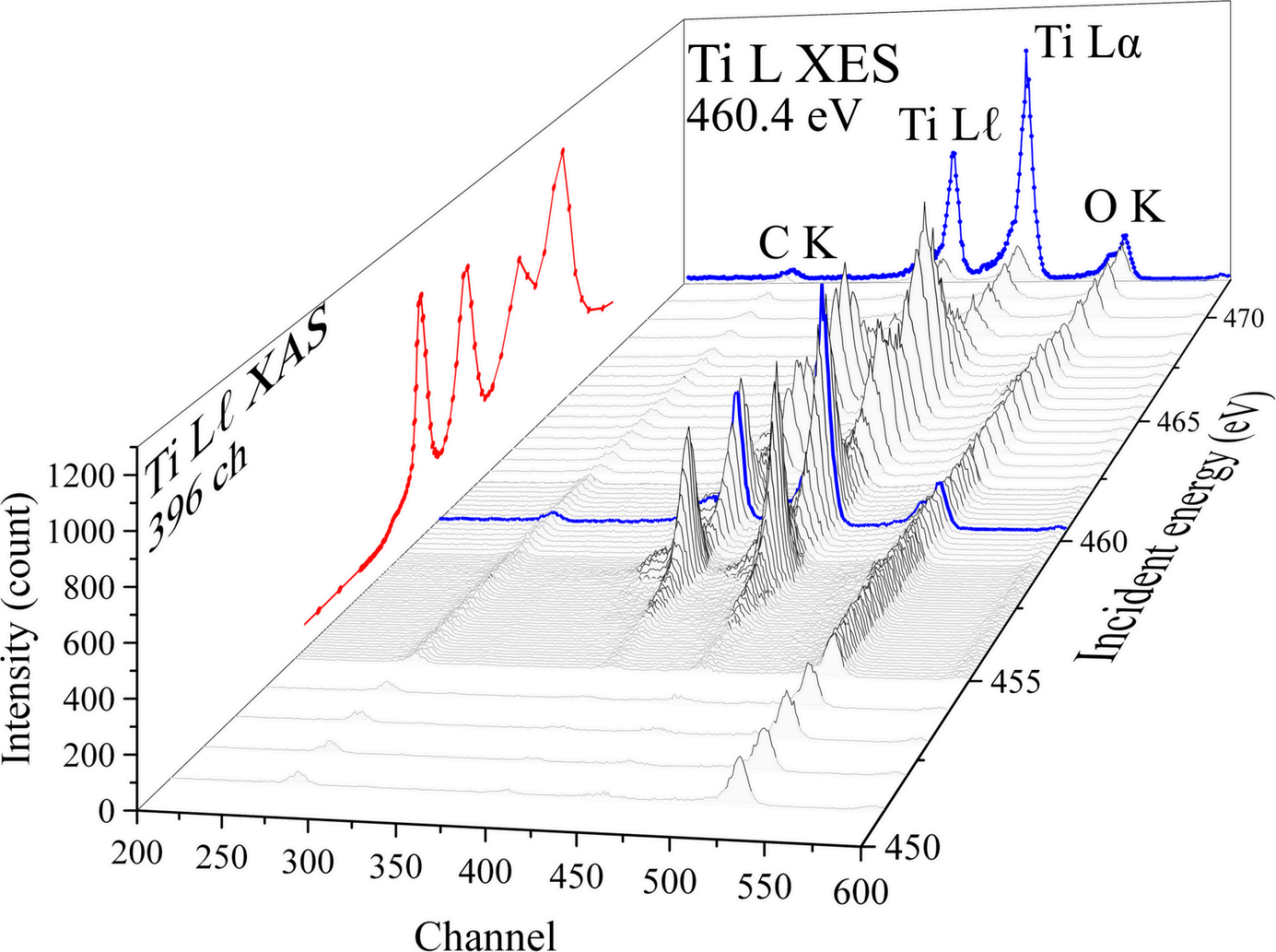
Birds-eye view of the performance of the STJ detector with incident X-ray energy, detection channel numbers and intensity. A fluorescence spectrum at an incident energy of 460.4 eV at the rear wall (blue line) can be seen, along with the PFY spectrum (red line) derived from the Ti *L*ℓ fluorescence line (channels 369–428) at the left wall.

Fig. 1 ▸ provides a comprehensive view of the performance of the STJ detector. The data are presented as a three-dimensional map of the fluorescence spectra depending on the incident X-rays energy. The map consists of three axes: incident X-ray energy, detector channel number, and fluorescence intensity. For example, the rear wall of the figure displays a fluorescence spectrum at an incident energy of 460.4 eV (blue line), corresponding to X-ray emission spectroscopy (XES). Both Ti *L*α and *L*ℓ fluorescence lines were simultaneously observed, and each exhibited sufficient intensity for analysis. This demonstrates that the STJ detector possesses the energy resolution required to distinguish two absorption edges located at closely spaced excitation energies in the soft X-ray region. In addition, a weak peak at approximately channel 278 was observed due to the residual carbon within the vacuumed chamber. The peak around channel 524, corresponding to the oxygen *K*-edge, was observed at an energy exceeding that of the incident X-rays. This feature is considered to result from excitation by second-order light of the incident beam.

A PFY spectrum is obtained by integrating these fluorescence lines within a specific detector channel width. As shown on the left side of Fig. 1 ▸, the PFY spectrum (red line) was derived over the Ti *L*ℓ fluorescence line (channels 369–428 ). Similarly, XAS spectra based on the Ti *L*α line are also acquired, enabling the simultaneous extraction of multiple PFY spectra from a single measurement. Thus, it is not necessary to predefine the energy window prior to data acquisition. Instead, the specific fluorescence line for analysis can be selected after the measurement.

### 3.2. Ti *L*-edge XAS

**Figure 2:**
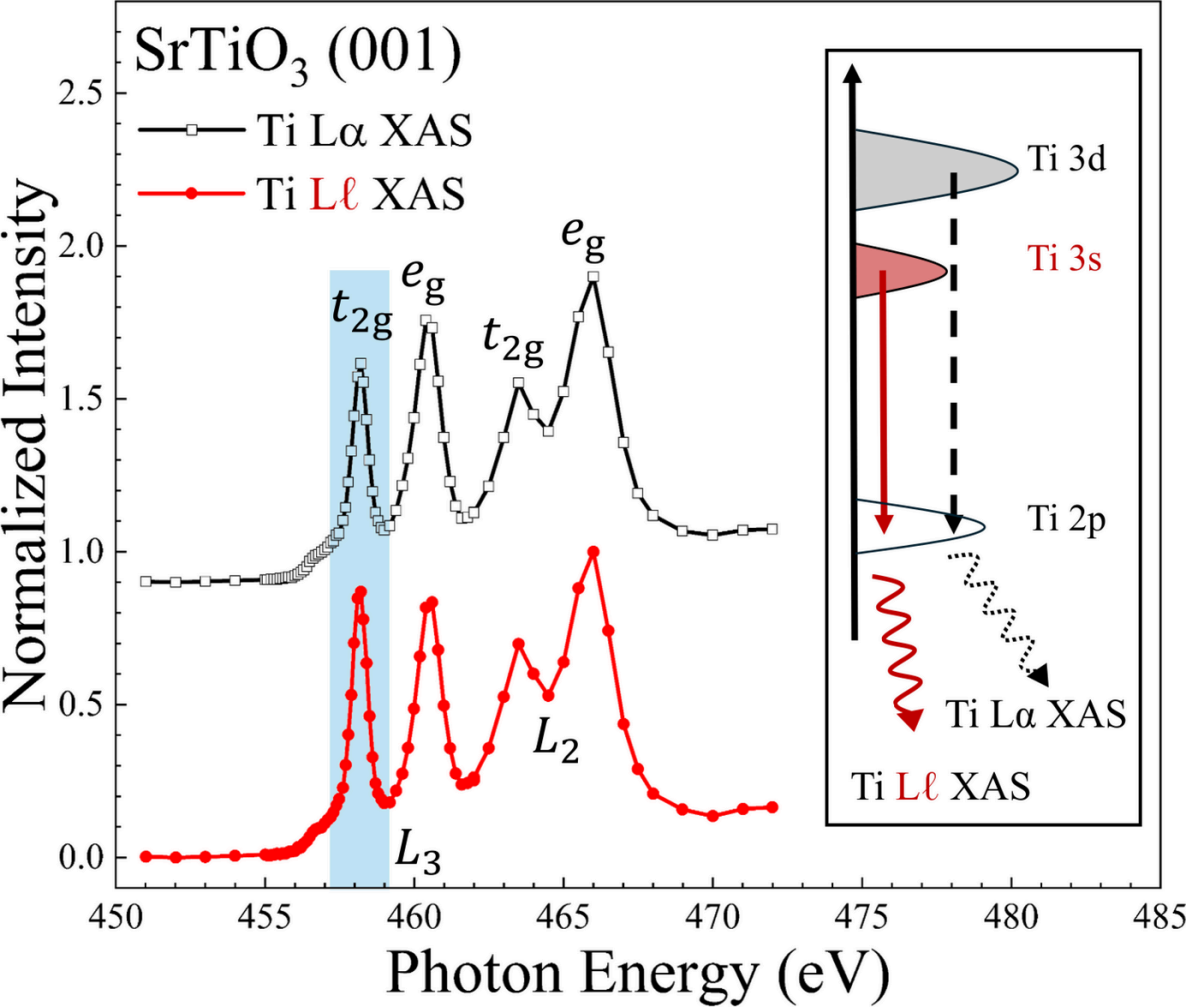
Comparison of Ti *L*ℓ (red filled circles) and Ti *L*α (black open squares) detection X-ray absorption spectra. The *L*_3_-*t*_2g_ peak of the former spectrum enhances as indicated by a blue frame. The inset shows a schematic energy diagram of the corresponding fluorescence lines.

Fig. 2 ▸ presents the XAS spectra obtained by electing two distinct fluorescence lines: Ti *L*α (black open squares) and Ti *L*ℓ (red filled circles). In both spectra, four peaks are observed; they arise from the spin–orbit splitting into *L*_3_ and *L*_2_, each of which is further split into *t*_2g_ and *e*_g_ by the crystal field (Burns, 1993 ▸). The inset of the figure schematically illustrates the electronic transitions responsible for the emission of Ti *L*α and *L*ℓ fluorescence X-rays. The former is attributed to de-excitation from the 3*d* to 2*p* orbitals, and the latter to de-excitation from the 3*s* to 2*p* orbitals. Conventionally, the Ti *L*α XAS is the standard for PFY-XAS at the *L*-edge. However, it is strongly affected by the self-absorption effect (3*de*_g_ → *t*_2g_ intra-atomic transition) (Chiu *et al.*, 2021 ▸). In contrast, the Ti *L*ℓ XAS is significantly less influenced by the electronic correrations, thereby yielding a more reliable and non-distorted spectrum (Miedema & Beye, 2018 ▸; Busse *et al.*, 2020 ▸; Chiu *et al.*, 2021 ▸).

A comparison of the two spectra, Ti *L*α XAS and *L*ℓ XAS, reveals that the intensity of the *L*_3_-*t*_2g_ peak in the Ti *L*ℓ XAS is enhanced relative to that in the Ti *L*α XAS (highlighted by a blue frame), while the other spectral features remain unchanged. This can be attributed to spatial distortion in the electronic orbitals; the Ti 3*d* orbitals have a preferred directional distribution, whereas the 3*s* orbitals are isotropic. This distinction is crucial for discussions of electronic properties, particularly those involving anisotropic electronic states.

### 3.3. *Medium*-energy-resolution fluorescence detected XAS

**Figure 3:**
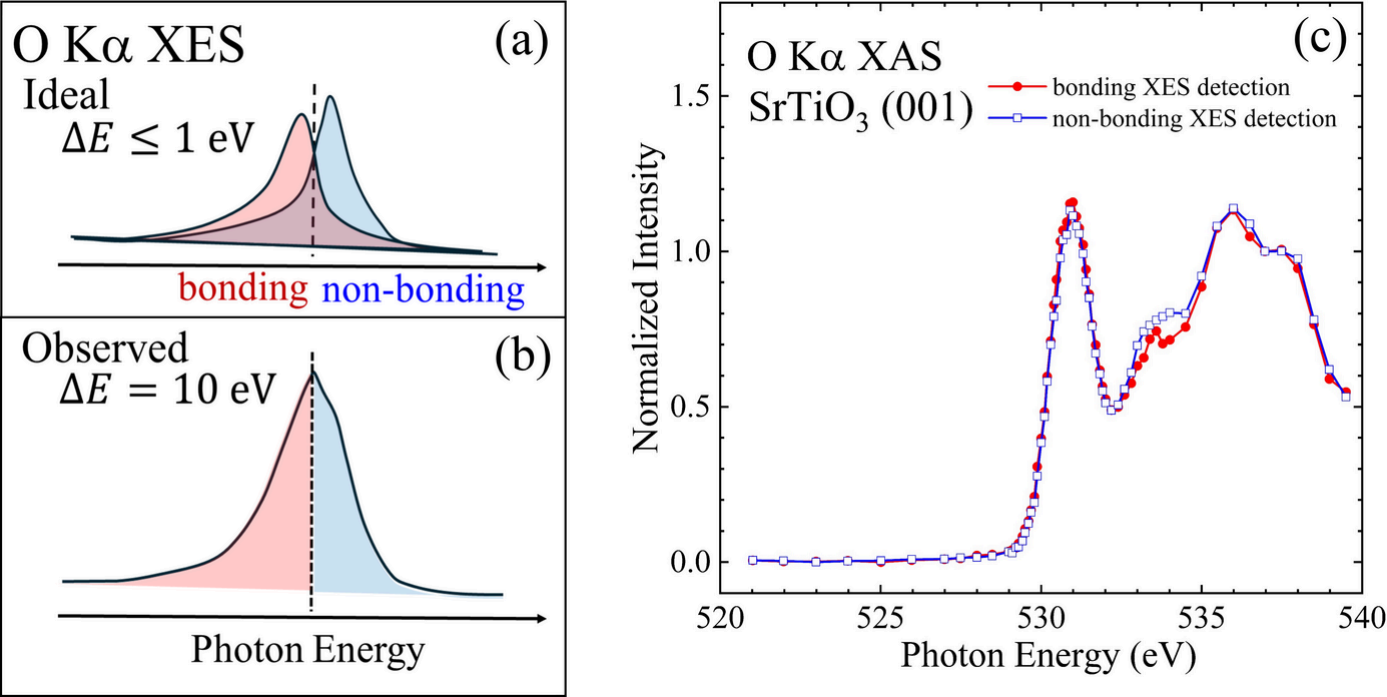
Fluorescence-line-selective oxygen *K*-edge XAS. The red/blue (filled circles/open squares) spectrum represents the lower/higher fluorescence-line detection, which corresponds to the fluorescence from bonding/non-bonding band.

It is important in the study of perovskite dielectric oxides to understand the oxygen 2*p* electronic states hybridized with the Ti 3*d* orbitals. Previous investigations have shown that the O *K*-edge XES exhibits a characteristic two-peak structure originating from the bonding and non-bonding bands (Nakajima *et al.*, 2010 ▸). A schematic illustration of the idealized emission spectrum is shown in Fig. 3 ▸(*a*). The lower and higher energy peaks correspond to the bonding and non-bonding bands, respectively. Their separation typically requires a detector with high energy resolution. Fig. 3 ▸(*b*) shows the O*K*-edge XES spectrum obtained in the present study. Owing to the limited energy resolution of the STJ detector, the bonding and non-bonding components are not fully resolved and convoluted.

Even though the energy resolution is not sufficient, we considered the fluorescence lines to the left-half and right-half of the O *K*α line originating from the dominant de-excitation states of the bonding and non-bonding bands, respectively, and the XAS spectra were obtained from each fluorescence line. The resulting spectra are compared in Fig. 3 ▸(*c*). A faint but discernible intensity difference at 534 eV is observed by comparing the two spectra. The reproducibility of the observed difference was confirmed in different beam times. Based on established trends in transition-metal oxides, the first peak at 531 eV is thought to originate from the *pd*π* hybridized orbitals between Ti 3*d* and oxygen 2*p*. Therefore, the structure at 534 eV is due to *pd*σ* hybridization (Wu *et al.*, 1997 ▸; Calandra *et al.*, 2012 ▸; Frati *et al.*, 2020 ▸). This is thought to reflect the anisotropic bonding state between Ti and its coordinating oxygen atoms. Experiments using SrTiO_3_ thin films are planned to further examine this anisotropy.

We would like to emphasize that, beyond the physical origin, the extraction of XAS spectra with selected de-excitation initial state becomes possible by selecting fluorescence X-rays from the same experimental data. This means that *medium*-energy-resolution fluorescence detected XAS can be conveniently achieved using an STJ detector.

## 4. Discussion

X-ray absorption spectra were measured at the Ti *L*-edge and O *K*-edge of SrTiO_3_ using the STJ detector. The STJ detector offers several notable advantages: it does not require a large-scale emission spectrometer, it enables simultaneous detection of multiple absorption edges in a single acquisition, and it provides flexibility in post-data analysis. In addition, high statistical precision can be achieved even on a bending-magnet beamline, thereby securing longer beam time and enabling effective systematic researches. Furthermore, the STJ detector enables XAS measurements under applied fields, electric or magnetic fields, making it well suited for the investigation of practical devices.

Generally, experimental verification have been less reported for electronic states of anions due to strong absorption in the soft-X ray region as well as the requirement for high energy resolution. We assure that a novel approach to the soft X-ray XAS using an STJ detector will resolve its difficulties and pioneer experimental investigation on the electronic states of anions.

## 5. Conclusion

We have successfully performed fluorescence-line-selective soft X-ray absorption spectroscopy on SrTiO_3_ using an STJ detector. The STJ detector offers excellent energy resolution and high detection efficiency, and flexiblity in data analysis. Simultaneous acquisition of the Ti *L*α and *L*ℓ XAS enabled us to obtain undistorted *L*-edge spectra free from orbital anisotropy effects. In addition, XAS measurements were achieved that reflect the bonding and non-bonding band states at the O *K*-edge. The results indicate that the STJ detector is a powerful tool for investigating the electronic states of anions in complex compounds such as carbides, nitrides and oxides.

## Data Availability

Data supporting this publication are available upon reasonable request to the corresponding author.
